# Magnetoresistance Effect and the Applications for Organic Spin Valves Using Molecular Spacers

**DOI:** 10.3390/ma11050721

**Published:** 2018-05-03

**Authors:** Xiannian Yao, Qingqing Duan, Junwei Tong, Yufang Chang, Lianqun Zhou, Gaowu Qin, Xianmin Zhang

**Affiliations:** 1 Key Laboratory for Anisotropy and Texture of Materials (Ministry of Education), School of Material Science and Engineering, Northeastern University, Shenyang 110819, China; 1670523@stu.neu.edu.cn (X.Y.); 1670385@stu.neu.edu.cn (Q.D.); 16700542@stu.neu.edu.cn (J.T.); qingw@smm.neu.edu.cn (G.Q.); 2 Computer Teaching and Researching Section, Shenyang Conservatory of Music, Shenyang 110818, China; changyf537@126.com (Y.C.); 3 Suzhou Institute of Biomedical, Engineering and Technology, Chinese Academy of Sciences, Suzhou 215163, China; zhoulq@sibet.ac.cn (L.Z.); 4 Northeastern Institute of Metal Materials Co., Ltd., Shenyang 110108, China

**Keywords:** organic spintronics, organic semiconductors, magnetoresistance effect, organic spin valves

## Abstract

Organic spin devices utilizing the properties of both spin and charge inherent in electrons have attracted extensive research interest in the field of future electronic device development. In the last decade, magnetoresistance effects, including giant magetoresistance and tunneling magnetoresistance, have been observed in organic spintronics. Significant progress has been made in understanding spin-dependent transport phenomena, such as spin injection or tunneling, manipulation, and detection in organic spintronics. However, to date, materials that are effective for preparing organic spin devices for commercial applications are still lacking. In this report, we introduce basic knowledge of the fabrication and evaluation of organic spin devices, and review some remarkable applications for organic spin valves using molecular spacers. The current bottlenecks that hinder further enhancement for the performance of organic spin devices is also discussed. This report presents some research ideas for designing organic spin devices operated at room temperature.

## 1. Introduction

Organic spintronics is an emerging research field, within the broader field of spintronics and organic semiconductors (OSCs). The magnetoresistance effect (MR) was firstl reported in organic spin valves (OSVs) [1,2]. The addition of spin freedom in spintronics greatly enriched the research content of microelectronics, because these new devices utilized not only electron charge transport, but also carrier spin transport [3,4]. OSCs have been extensively explored, mainly because their high structural flexibility, low production cost, and large area coverage [5,6,7,8,9,10,11,12,13]. In particular, OSCs are expected to feature a long spin life based on the weak spin-orbit coupling (SOC) strength and weak hyperfine interaction (HFI) [14,15,16,17,18,19,20,21,22], which contribute to the development of next generation nano-electronic devices [1,2,23,24,25,26,27,28,29].

To date, OSV is one of the most popular devices for investigating spin dependent transport in OSCs. A general OSV has a sandwich structure with two ferromagnetic (FM) electrodes (with different coercive fields) which are separated by a nonmagnetic organic layer. A common way to detect organic-based spin dependent transport is the electrical measurement of magnetoresistance in these spin-valve structures. The resistance of the device can be switched by sweeping an external magnetic field, leading to a magnetic alignment rotation of the ferromagnetic layers, from parallel to antiparallel. Usually, a higher (lower) resistance can be observed when the magnetization directions are antiparallel (parallel). Generally, the MR effect can be divided into giant magetoresistance (GMR) [30,31] and tunneling magetoresistance (TMR) [32,33]. GMR is a quantum mechanical effect which was first observed in Fe-Cr multilayers. TMR is a magnetoresistive effect that normally takes place in a magnetic tunnel junction (MTJ) with a very thin insulating layer. Both have revolutionized the field of magnetic sensors, magnetic storage, and information recording, and have also been used in OSVs. For instance, TMR devices usually feature MR values with zero residual time for both charges and spins in the organic barrier. This enables them serve as logic and magnetic sensitive devices. However, in GMR devices, a finite spin lifetime [15,34] in OSCs during injection and detection can allow for spin manipulation. This makes it possible for such devices to magnetically control organic light-emitting diodes (OLEDs) [35,36,37,38], organic photovoltaic devices [39,40], and even quantum computing systems [6,41,42]. In this review, we introduce the basic knowledge required to fabricate and evaluate organic spin devices. Progress in spin transport investigations, for both GMR and TMR devices using small organic molecules as space layer, has been made over the last decade. The current bottlenecks that hinder further performance enhancement in organic spin devices is also discussed.

## 2. Spin Polarization of Ferromagnetic Electrode 

**Figure 1:**
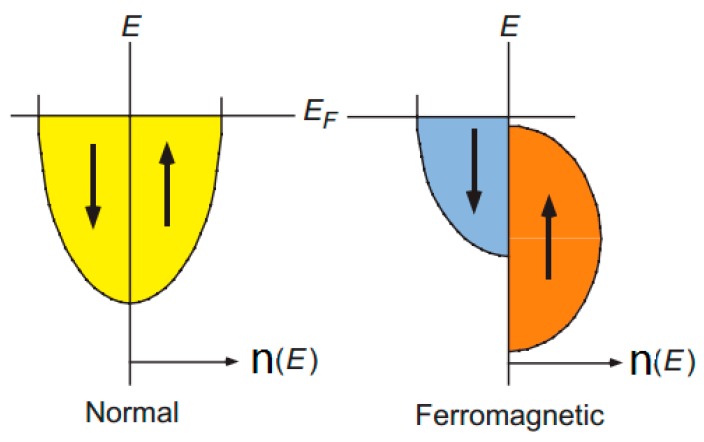
A schematic of the density of electronic states in normal and ferromagnetic metals. The density is equal for the normal metal, and imbalanced for the ferromagnetic. E is the electron energy; E_F_ is the Fermi energy level; n (E) is density of states. Adapted from [3], with permission from © 1998 The American Association for the Advancement of Science.

Coherent tunneling and incoherent hopping are two widely accepted cases of the conduction regime in the OSVs [16,28,43], which correspond to the TMR and GMR effects in the OSVs respectively. In the case of the TMR, electrons can tunnel directly from one magnetic electrode into the other via a thin barrier (within several nanometers). The effect depends on the magnetization and spin polarization (P) of two ferromagnetic electrodes. P is defined in terms of the number of carriers n that have spin-up (↑) and spin-down (↓), thus P = (n↑ − n↓)/(n↑ + n↓). The imbalance of spin-up (↑) and spin-down (↓) electrons at the Fermi level in ferromagnetic metals naturally leads to an inequality of spin density, which can produce a net spin polarization, as shown in Figure 1 [3]. The TMR effect can be expressed by the following equation [44]:

(1) $$\mathrm{TMR}=\frac{2P_{1}P_{2}}{1-P_{1}P_{2}}$$

P_1_, P_2_ is denoted as the spin polarization of each electrode. Generally, the high spin polarization of FM electrodes contributes to high TMR response. Some potential ferromagnetic materials for electrodes in spin valves are listed in Table 1. Although the Curie temperature is very high for 3d transition metals (Fe, Co, Ni) and their alloys, the electrons are usually not fully spin polarized at the Fermi energy level, leading to a low spin polarization. In contrast, FM materials such as La_0.67_Sr_0.33_MnO_3_ (LSMO), CrO_2_, Fe_3_O_4_, and Co_2_MnSi show a high spin polarization because their electrons are nearly full polarized at the Fermi energy level. As summarized in Table 1, the Curie temperatures for LSMO and CrO_2_ are near to room temperature. The Curie temperatures for Fe_3_O_4_ and Co_2_MnSi are beyond 800 K, indicating a great potential as electrodes for spin transport in spin devices operated at room temperature [45,46].

For the thicker organic molecular layer, the one-step tunneling is no longer dominant where the electrons transport occurs by diffusion or hopping [43], resulting in the GMR effect. Spin carriers propagate by random site-to-site hopping between pseudo localized states at both intra- and inter-molecules [16]. Similarly, spin polarization of FM electrodes is decisive in the efficiency of spin injection and detection in GMR devices.

## 3. Traditional Organic Spin Valves

### 3.1. GMR and TMR Effects in Molecule Spin Valves

**Figure 2:**
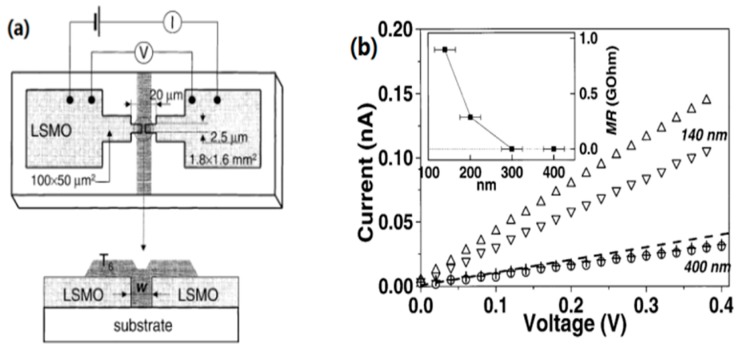
(**a**) The schematic of the LSMO/T_6_/LSMO lateral spin-valve device. (**b**) I-V characteristics of LSMO/T_6_/LSMO as a function of magnetic field. Down triangles and circles correspond to H = 0 Oe, while up triangles and crosses to H = 3.4 kOe. The inset indicates MR as a function of the channel length of T_6_. Adapted from [2], with permission from © 2002 Elsevier.

Typically, OSVs can be classified as lateral structure devices and vertical structure devices, based on the device structure. In Figure 2a, Dediu et al. have reported a LSMO/T_6_/LSMO lateral spin valve, which was considered as the first communication on spin injection on OSCs. In Figure 2b, the MR showed a strong decrease with increasing T_6_ channel length at 100–200 nm. The spin diffusion length in T_6_ was estimated at about 200 nm at room temperature [2]. Ikegami et al. also reported a LSMO/pentacene/LSMO lateral spin valve [59]. The space between the electrodes was in the range of 50 to 300 nm. It was found that the MR ratio depends on the gap distance of the electrodes. A MR ratio nearly of 6% was observed at 5.3 K, and the spin diffusion length was estimated to be at least 55 nm in pentacene. The spin diffusion length in the organic system scatters over a large scale, and will be discussed in the following section.

**Figure 3:**
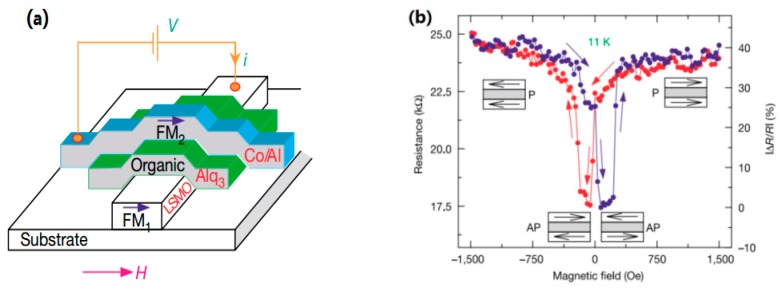
(**a**) Schematic diagram of a vertical spin valve consisting of a bottom LSMO electrode and a top Co electrode with the Alq_3_ spacer. (**b**) Negative MR up to 40% in spin valve with 130 nm Alq_3_ at 11 K. The blue (red) curve represents GMR measurements made with increasing (decreasing) magnetic field H. AP and P was dubbed as the magnetization of two FM electrodes. Adapted from [1], with permission from © 2004 Springer Nature.

Xiong et al. [1] first fabricated a vertical spin valve with an LSMO/Alq_3_/Co structure, shown in Figure 3a. A bottom electrode LSMO (100 nm) and a top electrode Co (3.5 nm) were separated by a thick Alq_3_ spacer (between 130 to 250 nm). MR = (R_AP_ − R_P_)/R_AP_, where R_AP_ and R_P_ denote as the resistances of the whole device when the two electrodes are in antiparallel and parallel magnetization. A negative GMR of up to 40% was observed at 11 K with 130 nm organic layer, using a four probe method. The GMR decayed with increasing temperature and vanished at 300 K, which demonstrates a strong dependence on temperature. It is worth noting that GMR in this system also showed dependence on bias voltage and thickness. However, the GMR signal is anomalous, that is, the antiparallel magnetization showed a lower resistance, which was attributed to the negative spin polarization of Co. In traditional OSVs, the materials of organic spacer layers are of essential importance for enhancing the MR response and the spin polarization of FM electrodes (see Table 1). Some representative molecule semiconductors and the combined electrodes have been summarized in OSVs, as listed in Table 2.

**Figure 4:**
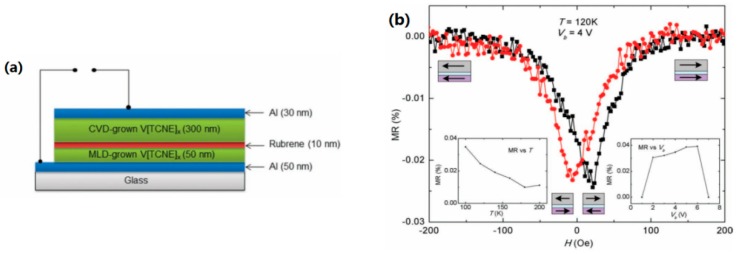
(**a**) A schematic diagram of all organic spin valve consisted of Al/V[TCNE]_x_/rubrene/V[TCNE]_x_/Al. The bottom and top Al serve as electrodes. (**b**) The observed MR as a function of varied magnetic field at 120 K and 4 V bias voltage. The insets showed the dependence of MR on both temperature and bias voltage. Adapted from [73], with permission from © 2011 John Wiley and Sons.

In the hopping regime in an OSC, the spin diffusion length λs and spin relaxation time τ can be related via a carrier diffusion coefficient D, λ_s_ $=\sqrt{D\tau }$ [60,61,62]. Additionally, D = *k*_B_Tμ/e; this has been established in both theories and experiments. *k*_B_, T and μ are the Boltzmann constants, absolute temperature and carrier mobility, respectively. In principle, higher carrier mobility contributes to a higher MR response. However, the carrier mobility in amorphous OSCs usually ranges from ∼10^−6^ to 10^−2^ cm^2^V^−1^s^−1^ [63,64,65,66,67]. For example, Alq_3_ shows an electron mobility of μ = 2.5 × 10^−5^ cm^2^V^−1^s^−1^ [68,69]. The carrier mobility of OSCs could be significantly enhanced by improving the crystalline or single crystal growth. It is reported that the crystalline rubrene (C_42_H_28_) shows a hole mobility as high as 10 cm^2^V^−1^s^−1^ at room temperature in OLED and organic field effect transistors (OFETs) [68,70]. Hence, a high MR response in rubrene-based spin valves could be expected by increasing carrier mobility. A hybrid structure Fe/Al_2_O_3_/rubrene/Co with different rubrene thickness (vary from 4 to 18 nm) was first fabricated by Shim et al. [23]. A TMR value of about 6% was observed at room temperature and a spin diffusion length λ_s_ = 13.3 nm was estimated at 4 K among these amorphous rubrene. Inelastic transport by hopping through delocalized states in disordered films would lead to a flip in spins. A longer λ_s_ was expected in OSVs using crystallized rubrene as space layers [23,29,71]. In vertical spin valves, one major fabrication challenge is the presence of an “ill-defined layer”, when FM metallic atoms are directly deposited onto “soft” organics. The alumina layer was introduced as a buffer layer between FM metal and organic semiconductor to enhance the spin injection at the FM/OSC interface [23,25,28,29,43]. The other challenge at the interface between FM and OSC is the conductivity mismatch [72,73,74,75]. To further reduce the conductivity mismatch at the interface between FM electrodes and OSC layer, Li et al. fabricated an all organic layer device with the V[TCNE]_x_/rubrene/V[TCNE]_x_ structure [73], as shown in Figure 4. However, no significant MR was obtained in this device, which may due to the poor spin polarization in organic magnetic materials. Nevertheless, this study provides a scenario for all future organic spin devices.

### 3.2. Effects of Spin Orbit Coupling and Hyperfine Interaction

The weak spin dependent scattering in the organic layer may be the most attractive aspect in organic spintronics [5,6,7,87]. SOC and HFI are two main factors that affect spin dependent scattering. SOC is the coupling between orbital angular momentum and spin momentum; the strength of SOC is proportional to Z^4^ (Z is the atomic number) [87,88,89,90]. Organic materials mainly consisting of C, H, O and N elements possess much lower atomic numbers than their inorganic counterparts; therefore, OSCs are expected to present a weaker spin orbit coupling. It is reported that SOC is indeed important for spin relaxation during spin hop in OSCs, such as tris-(8-hydroxyquinoline) aluminum (Alq_3_) [25,27,91] and copper phthalocyanine (CuPc) [78,92,93]. The spin relaxation mechanism in Alq_3_ was analyzed based on the Elliot-Yafet mechanism, thanks to the presence of SOC [34,94]. Nuccio et al. studied the influence of SOC strength on spin relaxation by substituting heavy atoms in the materials, indicating that SOC strength increases almost linearly with atomic numbers [95]. 

**Figure 5:**
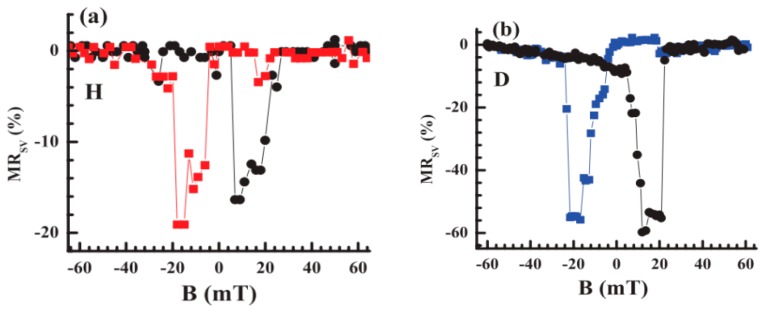
MR curves of two 40 nm thick OSV based on (**a**) H-Alq_3_ (**b**) D-Alq_3_, measured at V = 12 mV and T = 10 K. Adapted from [98], with permission from © 2015 American Physical Society.

The importance of HFI on MR response has been underlined in both theories and experiments [96,97,98,99]. HFI is the spin-spin interaction between nucleus spin and electron spin, which is principally affected by polarized hydrogen nuclei and other nuclear atoms in OSCs [90,99]. Nguyen et al. studied the magnetotransport in both H_18_Alq_3_ (protonated) and D_18_Alq_3_ (deuterated)-based spin valves [98]. As showed in Figure 5a,b, the MR ratio of a D_18_Alq_3_ spin valve is three times larger than that of an H_18_Alq_3_ based device, indicating that the spin diffusion length in D_18_Alq_3_ is substantially longer than that of H_18_Alq_3_. The difference originates from the isotope exchange in molecule spacer layer. The hydrogen atoms of Alq_3_ (nuclear spin I_H_ = 1/2, nuclear g factor g_H_ = 5.586) are replaced by deuterium atoms (I_D_ = 1, g_D_/g_H_ = 0.154). The HFI constant is in proportion to the g factor. Thus, spin transport is efficient in deuterated Alq_3_ devices with a smaller HFI, giving rise to in a higher MR response. Further theoretical investigations may be found in the literature [13,24,71,74,87,89,94,95].

**Figure 6:**
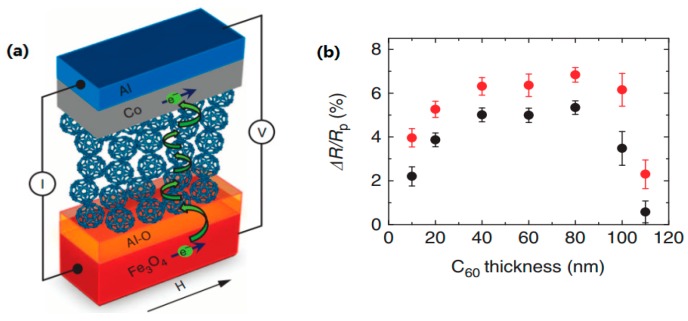
(**a**) A schematic diagram of Fe_3_O_4_/AlO*_x_*/C_60_/Co hetorejunction with a MgO substrate (not shown in the diagram). (**b**) Dependence of MR ratio on C60 thickness. The MR was measured at 300 K (black dots) and 150 K (red dots) under a 30 mV bias voltage. The error bars represent a standard deviation of different measured values at each thickness. Adapted from [28], with permission from © 2013 Springer Nature.

It is conceivable that a ^12^C atom is of nearly zero nuclear spin, implying the HFI is approximately zero, and therefore, negligible [100]. Due to the presence of isotope ^13^C nuclear spin, and the absence of polarized hydrogen nuclei, spin diffusion length in C_60_ is expected to be longer than that of other OSCs. Meanwhile, C_60_ is highly symmetrical and is nearly isotropic; these facts make it possible for spin polarized carrier hops from delocalized states within a small spin flip and with small energy loss. Zhang et al. [28] fabricated a hybrid structure Fe_3_O_4_/AlO*_x_*/C_60_/Co, as shown in Figure 6a. It is worth noting that that Fe_3_O_4_ is nearly fully spin polarized at Fermi level by density-functional calculation [53]. Particularly, Fe_3_O_4_ possesses a high Curie temperature at 860 K, which is much higher than that of LSMO. These characteristics make it possible to fabricate room temperature devices, even at high temperatures. Thus, a room temperature GMR valued 5.3% was observed and a long spin diffusion length up to 110 nm was estimated in C_60_. More interestingly, an unusual dependence of MR ratio on C_60_ thickness was observed, as shown in Figure 6b. Thickness dependence showed parabolic behavior, which was different from previous reports with other OSCs [1,101]. The GMR ratio at both 150 K and 300 K showed the maximum value for the devices with a C_60_ thickness of around 80 nm. It was concluded that the carrier mobility increases in an organic layer, and the electric field strength reduces with increasing C_60_ thickness. These combined factors result in the maximal value of MR ratios depending on C_60_ layer thickness. This special temperature dependence has been confirmed by Vardeny group at low temperatures [102].

### 3.3. Dependence of MR on Measurement Temperature

**Figure 7:**
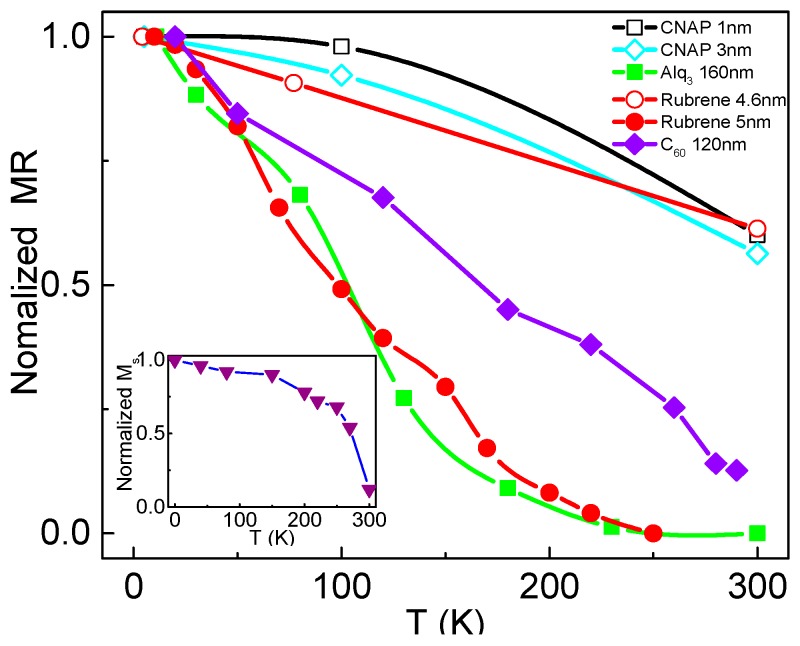
Normalized MR on temperature dependence measured by different groups, all of which decayed with increasing temperature. The hollow dots represent TMR, the solid dots represent GMR. (CNAP, Suzuki et al. [43]; Alq_3_ 160 nm, Xiong et al. [1]; Rubrene 4.6 nm, Shim et al. [23]; Rubrene 5 nm, Yoo et al. [104]; C_60_ 120 nm, Liang et al. [60].) The inset shows the magnetization of LSMO versus T, adapted from [1], with permission from © 2004 Springer Nature.

One of the critical goals for OSVs is to achieve an MR effect at room temperature. However, for most of the OSV devices reported to date, MR behavior is strongly suppressed by increasing measurement temperature [1,23,25,26,27,28,29,103]. MR dependence on temperature for several representative organic materials is listed in Figure 6. It was found that devices using CNAP as spacers with 1–3 nm show a slow decay with increasing temperatures. In contrast, devices using Alq_3_ and C_60_ as spacers, with the thickness over 100 nm, show a quick decay with increasing temperatures. Generally, the decay ratio of the TMR effect with increasing temperature is slower than that of GMR response. TMR effect would be dominant in devices using an organic spacer layer of several nanometers [23,25,43], and the GMR effect should be decisive in devices using an organic spacer layer of over tens of nanometers [1,60]. These results clearly show that the spin dependent transport mechanism for the devices with CNAP differs to that of Alq_3_ and C_60_ based devices. In addition, the TMR ratio largely depends on the spin polarization of device electrodes. As shown in Figure 7, Fe/rubrene/Co and LSMO/rubrene/Co with a close organic layer thickness (∼5 nm) show an obvious difference in MR dependence. This is very likely caused by a serious decrease in the spin polarization of LSMO with increasing temperature [1,60,76], as shown in the insert of Figure 7.

The coupling between the FM and OSC at the interface is crucial for spin injection. The interface is known as the spinterface, where the organic molecular orbitals and the electronic energy levels of FM are hybridized [76,105,106]. The signal of MR results can be opposite or negative, depending on the various spinterface. Barraud et al. explained that the formation of a spinterface cause a spin-dependent broadening of the localized states [107]. Cichetti at al. reported a spin injection efficiency of ∼90% from the unoccupied molecular orbitals of CuPc into the cobalt [92]. Spinterface effects have also been explored in theory to understand the effect of orbital hybridization [108,109]. Djeghlou et al. observed a highly spin-polarized interface between Co and phthalocyanine at room temperature, suggesting an exceptionally large MR response (up to 500%) [109]. 

**Figure 8:**
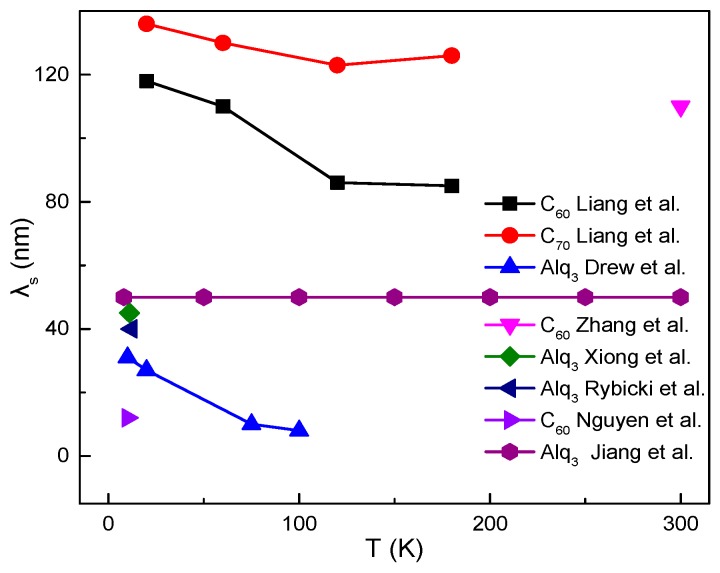
Spin diffusion length λ_s_ as a function of temperature. (C_60_, C_70_ Liang et al. [60]. C_60_ Zhang et al. [28]. C_60_ Nguyen et al. [102]. Alq_3_ Xiong et al. [1]. Alq_3_ Rybicki et al. [101]. Alq_3_ Drew et al. [91]. Alq_3_ Jiang et al. [112].)

As shown in Figure 8, the spin diffusion lengths (λ_s_) have revealed significant variations between different organic semiconductors. Moreover, even if the same OSC is used in spin devices, λ_s_ scatter on a large scale. This is probably due to the change of defect states appearing in OSC films prepared by different groups. Rybicki et al. proposed that the λ_s_ in Alq_3_ is very sensitive to the trap density [101]. In addition, different FM/OSC spinterfaces may also influence the efficiency of spin injection and detection, leading to the change of λ_s_. Voltage control of magnetism has been extensively investigated in non-organic spintronics. It is also reported that the spin manipulations in organic spacers can be accomplished by magnetic and electric fields [110,111]. Pramanik et al. [94] observed the spin valve signal in a Ni/Alq_3_/Co nanowire spin valve, and found that the signal decays with increasing bias current. This is probably due to the increased carrier scattering, leading to more rapid spin relaxation and a shorter spin diffusion length. Therefore, the voltage control of spin diffusion in the organic channel would be a potential avenue for further explorations.

### 3.4. Nanowire Spin Valve

**Figure 9:**
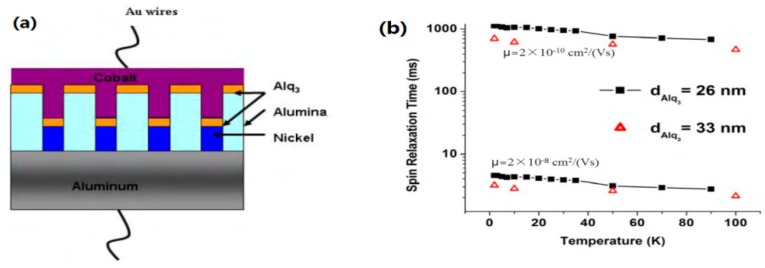
(**a**) A schematic diagram of nanowire spin-valve structure consisting of Co/Alq_3_/Ni. (**b**) The calculated spin diffusion time τ as a function of temperature at different carrier mobility μ. Adapted from [34], with permission from © 2007 Springer Nature.

The spin lifetime in OSC based devices is in the range of μs and up to s, which is of several magnitudes longer than that of inorganic based systems [15]. Pramanik et al. [34] fabricated a nanowire spin valve structure (see Figure 9a) consisting of Co/Alq_3_/Ni. They extracted the spin diffusion length from a modified Julliere model [1]:

(2) $$\mathrm{MR}=\frac{2P_{1}P_{2}e^{-\left(d-d_{0}\right)/\lambda_{s}}}{1-P_{1}P_{2}e^{-\left(d-d_{0}\right)/\lambda_{s}}}$$

where d is the thickness of the organic layer which can be monitored by a crystal oscillator, and can be verified by TEM. d_0_ is the thickness of the ‘ill-defined’ layer. Since d_0_ << d, the authors assumed that d − d_0_ ≈ d. P_1_ and P_2_ is the spin polarization of Co and Ni, respectively. The calculated λ_s_ is around 4∼6 nm. Hence, the spin relaxation time can be extracted by τ (T) = λ_s_^2^/D = eλ_s_^2^/k_B_Tμ. The calculated τ is showed as a function of T in Figure 9b. It is noted that τ is exceptionally long, close to a magnitude of seconds. This is very useful for the development of quantum computers by addressing spin degree of freedom of individual quantum dots within spin coherence time [6,7,41]. However, maintaining a long enough quantum spin coherence time and addressing high performance logical operations within this period is still the challenge. The coincidence in meeting the requirements is the extremely long spin life in organic based systems, which may enable the realization of quantum computing in organic spintronics.

### 3.5. Hanle Effect in OSVs

The Hanle effect has been widely used to assess the spin injection in the semiconductor spintronics. Riminucci et al. [113] fabricated a LSMO/Alq_3_/AlO*_x_*/Co vertical spin valve. They investigated the Hanle effect by measuring the GMR at different angles between the device’s plane and the magnetic field, and found no sign of its presence. Yu proposed that spin-charge decoupling suppresses Hanle effect and causes spin diffusion in OSCs [13]. Watanabe et al. [114] reported the observation of Hanle effect while measuring angular dependence of inversed spin Hall effect in polymers. Recently, Jiang et al. [105] studied spin transport mechanisms in a Y_3_Fe_5_O_12_/Alq_3_/Pd system, and found that the angular dependence of inversed spin Hall Effect is attributable to a spin exchange mechanism, rather than the Hanle effect. Thus, the Hanle effect in organic systems is still controversial and further studies maybe very worthwhile.

## 4. Other Spin Devices

### 4.1. Spin-OLED 

**Figure 10:**
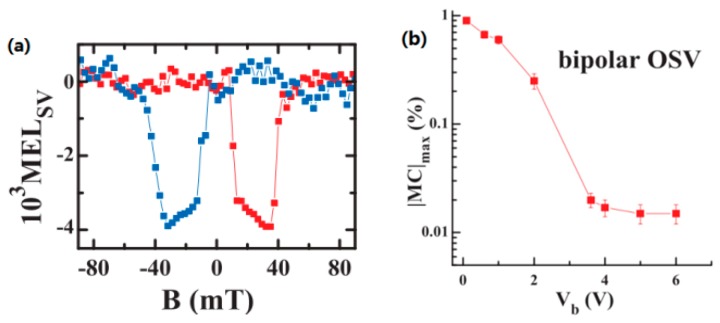
(**a**) A MEL loop after subtraction of the background in a typical spin-OLED device with organic layer thickness of 25 nm measured at V_b_ = 4.5 V and T = 10 K. The red (blue) loop stands for increasing (decreasing) external magnetic field. The switches of the MEL correspond to the coercive field of the FM electrodes. (**b**) The normalized maximum magneto-conductivity in bipolar devices as a function of bias voltage. Adapted from [35], with permission from © 2012 The American Association for the Advancement of Science.

One importantly potential application for organic spintronics is spin-OLED. In such a device, the electroluminescence (EL) intensity can be controlled by manipulating the mutual magnetization directions of spin injecting FM electrodes. In traditional OLEDs, the radiative recombination of both electrons and holes from the singlet excitons induces EL. This results in an upper limit quantum efficiency of 25% in statistics [115]. However, in OLED devices with FM electrodes, the formation of singlet states is enhanced when the magnetic configuration is antiparallel. This increases the quantum efficiency by up to 50% [88,116]. Nguyue et al. fabricated bipolar spin-OLED devices using an LSMO anode and a Co cathode [35]. Figure 10a shows a typical magnetic electroluminescence (MEL) loop after subtraction of background EL signal in a spin-OLED device. This indicates that the EL response is from the OSV device. The device shows ∼1% spin valve magneto EL response, as shown in Figure 10b. The magneto-conductivity decreased steeply for V_b_ < 3.5 V and leveled off while V_b_ > 3.5 V. This property can facilitate the realization of spin-OLEDs at a gate bias voltage V_b_ = 3.5 V. The result provides a pathway for organic displays controlled by external magnetic fields.

### 4.2. Spin-Photovoltaic Devices 

**Figure 11:**
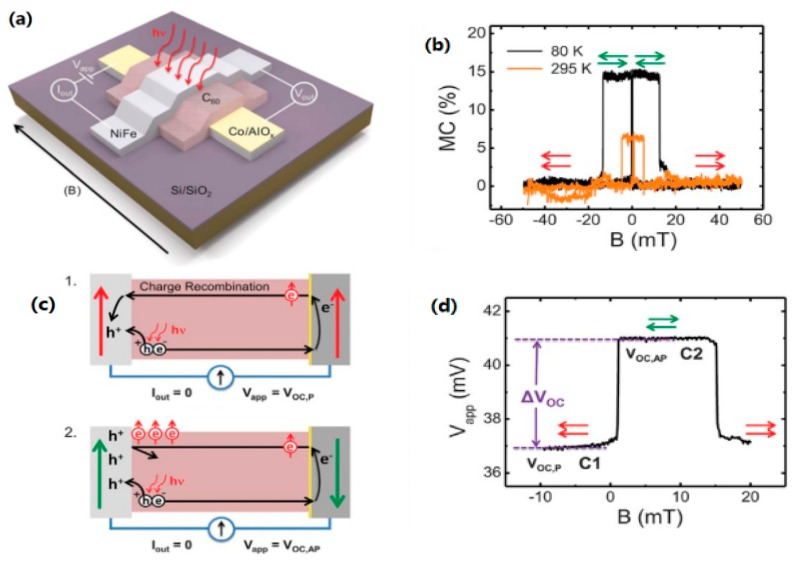
Illustration of a spin-photovoltaic device. (**a**) A schematic diagram of the spin-photovoltaic device. (**b**) The MC effect measured at 80 and 295 K with a bias of 10 mV in dark condition. MC = (I_P_ − I_AP_)/I_AP_ × 100%, where I_P_ and I_AP_ represent the parallel and antiparallel case of two electrodes. (**c**) Function principle of the spin-photovoltaic device in open circuit mode under an external magnetic field. (**d**) Bias voltage versus magnetic field at 80 K in open-circuit mode. Adapted from [117], with permission from © 2017 The American Association for the Advancement of Science.

Recently, Sun et al. fabricated a molecular spin-photovoltaic device [117]. The hybrid spin-valve with Co/AlO*_x_*/C_60_/Ni_80_Fe_20_ structure shown in Figure 11a illustrates a magnetocurrent (MC) effect of 15% at 80 K and of 6.5% at room temperature (see Figure 11b). The non-spin-polarized carriers generated by photovoltaic effect will not influence the MC with a switchable magnetic field. The open-circuit voltage VOC is defined as photogenerated bias at zero current. A spin photovoltaic response was confirmed in Figure 11c,d. In Figure 11c, spin polarized electrons from Co electrode transport through the C_60_ layer and arrive at the NiFe electrode when the magnetization configuration is parallel. Then, spin polarized electrons recombine with photogenerated holes, resulting in a low bias V_OC, P_. For the antiparallel magnetization, the collected holes in NiFe electrode could only be compensated by the injected spin-polarized electrons, resulting in a high bias V_OC,AP_, as shown in Figure 11d. The ΔV_OC_ = V_OC,AP_ − V_OC,P_ is the spin-photovoltaic response caused by spin polarized carriers accumulation at the spinterface. This study also presented a molecular spin photovoltaic device in light sensitivity and magnetic field controlled current inverter, which indicates a potential application for molecular spin optoelectronics.

## 5. Concluding Remarks

The organic nature of spin microelectronic devices could provide high structural flexibility, low production cost, and large area processing, making organic spintronics a promising alternative to conventional inorganic spintronics. However, it should be noted that the MR ratio in organic spin devices at room temperature was low compared to that of present inorganic spintronics. Meanwhile, maintaining a sufficient quantum spin coherence time and addressing high performance logical operations within this period are still challenges facing the development of quantum computers. This depends on the design of new materials for both efficient FM electrodes and OSVs. Moreover, the interface between FM and OSC significantly affects the spin scattering. A deep understanding and precise modification of the interface property will contribute to the development of spin devices with high performance operated at room temperature for future applications. In addition, the voltage control of magnetism has been extensively investigated in non-organic spintronics, owing to extremely low power consumption. An exploration of the spin manipulations in OSC molecule devices using an electronic field would be interesting for both fundamental science and future spin device development.

## Figures and Tables

**Table 1:** Representative FM materials used as OSV electrodes.

Electrode	Spin Polarization P (%)	Curie Temperature T_C_ (K)
Fe	44 [47]	1043 [48]
Co	34 [47]	1388 [49]
Ni	31 [50]	631 [48]
LSMO	100 [51]	369 [52]
Fe_3_O_4_	∼100 [53]	851 [54]
CrO_2_	100 [55,56]	392 [57]
Co_2_MnSi	100 [58]	900 [58]

**Table 2:** Organic molecules and FM electrodes used in spin valves and MR ratio.

Organic Materials	Chemical Structure	FM Electrodes	MR @ Temperature
T_6_	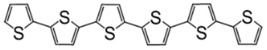	LSMO/LSMO [2]	30%@ RT [2]
Alq_3_	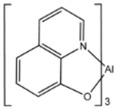	LSMO/Co [1] Co/Al_2_O_3_/Py [25] LSMO/Co [26,76]	−40%@11 K [1] 6.0@300 K [25] 300%@2 K [76] @10 K [26]
Rubrene	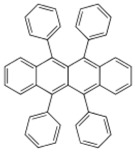	Fe_3_O_4_/AlO*_x_*/Co [29] Fe/Al_2_O_3_/Co [23]	6%@ RT [29] 16%@4.2 K 6%@295 K [23]
C_60_	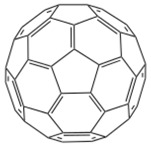	Fe_3_O_4_/AlO*_x_*/Co [28] Co/AlO*_x_*/Py [77]	5.3%@ RT [28] (5–10)%@ RT [77]
C_70_	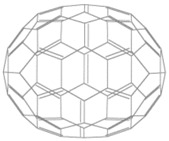	Fe_3_O_4_/AlO*_x_*/Co [78] LSMO/Co [60]	2.5%@150 K 0.3%@300 K [78] 6%@20 K 0.7%@290 K [60]
CuPc	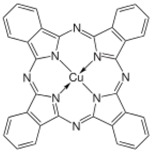	LSMO/Co [79] Co/AlO*_x_*/Ni_80_Fe_20_ [80]	6%@ 10 K 0.84%@ RT [79] >4%@ RT (F_16_CuPc) [80]
pentacene	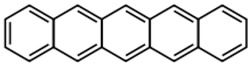	LSMO/LSMO [59,81]	2%@9 K [59] 5.5%@5.3 K [81]
PTCDA	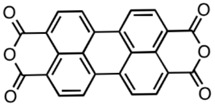	Fe/Co [82] NiFe/Co/AlO*_x_*/AlO*_x_*/Co [83]	0.4%@9 K [82] 13.5%@ RT [83]
α-NPD	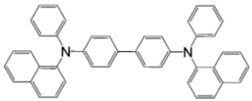	LSMO/Co [84]	14 ± 4%@14 K [84]
CVB	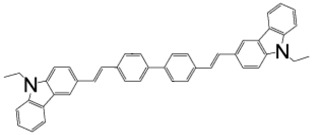	LSMO/Co [84]	18 ± 3%@14 K [84]
CNAP	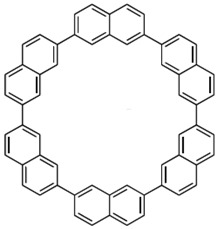	Co/AlO*_x_*/Ni_80_Fe_20_ [43]	4–6%@5 K 1–2%@300 K [43]
benzofurane bithiophene (BF3)	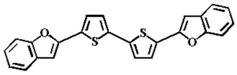	NiFe/AlO*_x_*/Co [85]	3%@40 K [85]
BCP	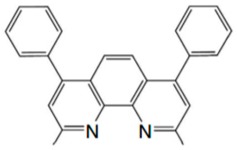	Co/AlO*_x_*/NiFe [86]	>4%@ RT [86]
TPD	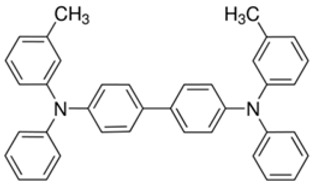	Co_2_MnSi/Co [58] LSMO/Co [58]	10.7%@5 K 7.8%@ RT [58] 19%@5 K [58]
